# Chloroform and Chloric Ether

**Published:** 1849-09

**Authors:** 


ART II.---*Effects of Chloroform and Strong Chloric Ether, as Narcotic Agents.*—By JOHN C. WARREN, M. D., &c., of Boston. Wm. D. Ticknor. 1849. pp. 66. 12 mo. (From the Publishers.)

Not long after the announcement that insensibility to the pain of Surgical operations, could be produced by Sulphuric Ether, various other substances were found to possess the same property. Amongst these the one that promised to be best adopted to the purpose, was chloroform. It was found to be certain and speedy; while, at the same time, its pleasant aromatic flavor, rendered it much less disagreeable than the ether. The narcotic properties of this powerful drug were laid hold of, not only by the

Surgeon, but by many who resorted to it for the purpose of enjoying the temporary ecstacy produced by it, and some for vile and crminal purposes. As might have been supposed, disastrous consequences, in many instances followed: the public became alarmed, and surgeons began to hesitate and reflect. Diligent search was made for other anæsthetic agents; and this little volume was written for the purpose of introducing to the profession Chloric Ether, which, however, is neither more nor less than diluted chloroform.

In this volume, Prof. W. has given detailed accounts of fatal results from the administration of chloroform, with the post-mortem appearances. We have not space at present to give our readers an analysis of the contents of this little volume, which we recommend to them as worthy of their attention. M

